# Supplementary material for: Modeling glioblastoma heterogeneity as a dynamic network of cell states
Source: Mol Syst Biol. 2021 Sep 16;17(9):e10105. doi: 10.15252/msb.202010105 (PMC8444284; doi:10.15252/msb.202010105)
Supplement: Supplementary file 5 — Source Data for Figure 3 [file MSB-17-e10105-s001.zip › Figure3A_sourcedata/GSEA_3065/nosigdb_state6.GseaPreranked.1622718443993/WENG_STEMNESS_PMID_30982771.html]

Details for gene set WENG\_STEMNESS\_PMID\_30982771[GSEA]

|  || Dataset | state6 |
| Phenotype | NoPhenotypeAvailable |
| Upregulated in class | na\_pos |
| GeneSet | WENG\_STEMNESS\_PMID\_30982771 |
| Enrichment Score (ES) | 0.5114824 |
| Normalized Enrichment Score (NES) | 1.7313517 |
| Nominal p-value | 0.0070422534 |
| FDR q-value | 0.008777467 |
| FWER p-Value | 0.167 |
Table: GSEA Results Summary

  

Fig 1: Enrichment plot: WENG\_STEMNESS\_PMID\_30982771      
 Profile of the Running ES Score & Positions of GeneSet Members on the Rank Ordered List

  

| PROBE | GENE SYMBOL | GENE\_TITLE | RANK IN GENE LIST | RANK METRIC SCORE | RUNNING ES | CORE ENRICHMENT || 1 | EEF1A1 |  |  | 9 | 0.325 | 0.0728 | Yes |
| 2 | PABPC1 |  |  | 15 | 0.292 | 0.1386 | Yes |
| 3 | CCND2 |  |  | 32 | 0.269 | 0.1977 | Yes |
| 4 | EIF4B |  |  | 68 | 0.231 | 0.2461 | Yes |
| 5 | SOX11 |  |  | 70 | 0.230 | 0.2983 | Yes |
| 6 | PTMA |  |  | 94 | 0.216 | 0.3448 | Yes |
| 7 | SET |  |  | 100 | 0.213 | 0.3926 | Yes |
| 8 | CORO1C |  |  | 345 | 0.145 | 0.3976 | Yes |
| 9 | HNRNPH1 |  |  | 378 | 0.139 | 0.4256 | Yes |
| 10 | TFDP2 |  |  | 507 | 0.122 | 0.4387 | Yes |
| 11 | H3F3B |  |  | 514 | 0.122 | 0.4657 | Yes |
| 12 | C6orf62 |  |  | 646 | 0.111 | 0.4758 | Yes |
| 13 | CD24 |  |  | 674 | 0.108 | 0.4973 | Yes |
| 14 | SOX2 |  |  | 863 | 0.093 | 0.4971 | Yes |
| 15 | TOX3 |  |  | 999 | 0.086 | 0.5011 | Yes |
| 16 | BAZ2B |  |  | 1208 | 0.075 | 0.4944 | Yes |
| 17 | TCF4 |  |  | 1234 | 0.074 | 0.5084 | Yes |
| 18 | CAMK2N1 |  |  | 1346 | 0.069 | 0.5115 | Yes |
| 19 | CHD7 |  |  | 1953 | 0.049 | 0.4534 | No |
| 20 | NFIB |  |  | 2762 | 0.028 | 0.3675 | No |
| 21 | ZBTB8A |  |  | 2887 | 0.024 | 0.3588 | No |
| 22 | TRA2A |  |  | 3410 | 0.013 | 0.3022 | No |
| 23 | TNFAIP8L1 |  |  | 3841 | 0.004 | 0.2540 | No |
| 24 | OPHN1 |  |  | 4021 | 0.000 | 0.2336 | No |
| 25 | FBLIM1 |  |  | 5182 | -0.022 | 0.1062 | No |
| 26 | TATDN3 |  |  | 5735 | -0.033 | 0.0507 | No |
| 27 | ZNF793 |  |  | 6290 | -0.044 | -0.0025 | No |
| 28 | PTPRS |  |  | 6439 | -0.048 | -0.0084 | No |
| 29 | HNRNPL |  |  | 6704 | -0.056 | -0.0258 | No |
| 30 | SRGAP2C |  |  | 6969 | -0.064 | -0.0415 | No |
| 31 | BOC |  |  | 7635 | -0.087 | -0.0976 | No |
| 32 | EGFR |  |  | 8251 | -0.126 | -0.1392 | No |
| 33 | RBM6 |  |  | 8413 | -0.148 | -0.1240 | No |
| 34 | ORC6 |  |  | 8482 | -0.157 | -0.0961 | No |
| 35 | MALAT1 |  |  | 8796 | -0.581 | -0.0000 | No |
Table: GSEA details [plain text format]

  

Fig 2: WENG\_STEMNESS\_PMID\_30982771: Random ES distribution      
 Gene set null distribution of ES for **WENG\_STEMNESS\_PMID\_30982771**
